# Dietary patterns and the risk of colorectal cancer and adenoma: a case control study in Iran 

**Published:** 2019

**Authors:** Alireza Bahrami, Mohammad Houshyari, Saeede Jafari, Pegah Rafiei, Mohammadreza Mazandaranian, Azita Hekmatdoost, Amir Sadeghi, Ehsan Hejazi

**Affiliations:** 1 *Student Research Committee, Department and Faculty of Nutrition Sciences and Food Technology, Shahid Beheshti University of Medical Sciences, Tehran, Iran*; 2 *Department and Faculty of Nutrition Sciences and Food Technology, Shahid Beheshti University of Medical Sciences, Tehran, Iran*; 3 *Department of Community Nutrition, Faculty of Nutrition Sciences and Food Technology, Shahid Beheshti University of Medical Sciences, Tehran, Iran *; 4 *Department of Clinical Nutrition and Dietetics, School of Nutrition Sciences and Food Technology, Shahid Beheshti University of Medical Sciences, Tehran, Iran*; 5 *Gastroenterology and Liver Diseases Research Center, Research Institute for Gastroenterology and Liver Diseases, Shahid Beheshti University of Medical Sciences, Tehran, Iran*

**Keywords:** Dietary pattern, Colorectal cancer, Colorectal adenoma, Healthy dietary pattern, Western dietary pattern

## Abstract

**Aim:**

The aim of this study is to examine the relationship between dietary patterns and the risk of Colorectal Cancer (CRC) and adenomas.

**Background:**

Dietary patterns have been shown to be associated with risk of CRC, but there are a few data on this context and its relationship with risk of colorectal adenomas as the precursors of the CRC.

**Methods:**

This hospital-based case-control study was conducted in three major general hospitals in Tehran province, Iran. Data was collected (October 2016 to May 2018) from 129 colorectal cancer and 130 colorectal adenoma patients that confirmed by pathology and colonoscopy findings and 240 controls with non-neoplastic conditions and not afflicted with diet related chronic diseases. Dietary data were evaluated by 147-items semi-quantitative food frequency questionnaire. Multivariate logistic regression was used to estimate the relationship between dietary patterns and risk of colorectal cancer and colorectal adenoma.

**Results:**

Three dietary patterns (healthy, western and traditional) were derived. After adjusting for confounders, the Healthy dietary pattern was associated with a decreased risk of Colorectal Cancer (OR=0.22, 95% CI=0.14-0.37) and Colorectal Adenoma (OR=0.43, 95% CI=0.27-0.69). Higher intake of the Westernized pattern was positively associated with risk of Colorectal Cancer (OR=3.5, 95% CI=2.13-5.19) and Colorectal Adenoma (OR=2.47, 95% CI=1.49-4.08). There was no significant association between traditional pattern and the Colorectal Cancer (OR=99, 95% CI=0.61-1.59) and Colorectal Adenoma (OR=0.85, 95% CI=0.54-1.35) risk.

**Conclusion:**

Our study suggested that the “Healthy” dietary pattern reduces the risk of Colorectal Cancer and Colorectal Adenoma, while the “Western” dietary pattern increases the risk of both Colorectal Cancer and Colorectal Adenoma.

## Introduction

 Colorectal Cancer (CRC) is one of the most common cancer that annually affect 1.2 million people in the world (1). This malignancy was reported the second most commonly diagnosed cancer in men and the third most commonly diagnosed cancer in women (2). In Iran CRC is the fourth most common cancer in men after stomach, bladder, and prostate cancers, and the second most common cancer in women after breast cancer, and an incidence of this cancer is reported to be 7-8 cases per 100,000 person (3, 4). It is well known that CRC is a multifactorial disease involving genetic and environmental (i.e. smoking, physical activity, alcohol intake and dietary habits) factors (5). Over the past decades, many studies have investigated the association of dietary factors and risk of CRC. Higher intake of vegetables and dairy products has been associated with decreased risk of CRC, whereas diet rich in red and process meat is related to an increased risk of CRC (6, 7). Most studies focus on individual foods and nutrients (8, 9), while in reality people consume meals containing combination of foods and various nutrients that are interactive or synergistic with each other (10). In this context, the examination of dietary patterns as a complementary approach for clarifying the relationships between diet and disease has been used (11). A number of studies have used dietary pattern analysis to investigate association between diet and risk of CRC (12-15), but most of them conducted in western and developed societies. On the other hand, despite Colorectal Adenoma (CRA) as known precursors of CRC (16). Few studies have investigated the relationship between dietary factors and CRA (17, 18) and there is a lack of published data on the topic of the relationship between dietary factors and the risk of colorectal adenomas in the middle-east region, specially Iran as a developing country. So, the aim of this study is to examine the relationship between Dietary Patterns and the risk of CRC and CRA. 

## Methods

**Subjects**

We conducted a hospital-based case-control study in three major general hospitals (Ayatollah Taleghani, Imam Hussein, Shohadae Tajrish) in Tehran province, Iran. CRC cases were patients with pathologic confirmation and colonoscopy findings, aged 30-79, diagnosed no longer than three months before the interview and had no history of cancers of other sites and previous diagnosis of Adenomatous polyp. CRA cases were “asymptomatic” individuals referred by their physicians for routine screening, rectal bleeding, or as a follow-up for positive routine fecal occult blood testing. The patients who had colorectal polyps as revealed in the colonoscopy and with histologically confirmed adenomatous polyp were assigned. Controls were selected randomly from patients admitted to the same hospitals as cases at the same time and same setting, with non-neoplastic conditions, not afflicted with diet related chronic diseases and aged 30-79. Controls were frequency matched on Age (±10 years) and Sex. Of 536 patients (268 control, 134 CRC, 134 CRA) selected for study, based on inclusion and exclusion criteria, patients with incomplete food frequency questionnaire and total energy intakes were outside the range of ±3 standard deviation from the mean, 28 controls, 5 CRC and 4 CRA were excluded.

**Assessment of dietary intake**

Participants’ dietary intake during the year prior to diagnosis was assessed for cases or controls in a personal interview using a valid and reliable semi-quantitative 147 food item food frequency questionnaire (FFQ)(19). Participants were asked to specify their consumption frequency for each food item on a daily, weekly, monthly or yearly basis. Intakes were then converted to daily frequencies and a manual for household measures was used to convert intake frequencies to daily grams of food intake (20). Energy and nutrient content of foods were calculated by the United State Department of Agriculture(USDA) food composition table (21). For some traditional Iranian food items that are not included in the USDA database (e.g., traditional breads), the Iranian food composition table was used. Due to Iranian religious beliefs, alcohol consumption was not asked, and therefore it was unavailable to the analysis. To identify dietary patterns, the 147 food items were categorized into 29 food groups (Table 1) based on their similarity of nutrient content and culinary usage or their relationship with cancer (15). Food items that were not fit to be included in a certain food group or were assumed to represent individual dietary behaviors were left as unique food groups (e.g. French fries, egg, and tea).

**Table 1:** Food groups used in dietary pattern analysis

Food group	Food items
Processed meat	Sausages, hamburger, salami
Red Meat	Beef, mutton, ground meat ,Visceral meat
Fish	Tuna, any type of fish
Poultry	Chicken
Egg	Fried eggs, boiled eggs
Low fat dairies	Low fat milk, Low fat yogurt, ordinary yogurt
High fat dairies	Whole milk, yogurt( high fat, drained and cream), cream cheese, cream, ice cream
Tea	Black tea
Coffee	Coffee
Fruits	Cantaloupe, watermelon, melon, sloe, apple, apricot, cherry, sour cherry, fig, nectarine, peach, pear, Citrus fruit, date, kiwi, grape, pomegranate, strawberry, banana, grape fruit, plum, persimmon, raisin, mulberry, compotes, other fruits
Artificial juice	packed juice
Tomato	Tomato
Carrot	Carrot
vegetables	Spinach, lettuce, mixed vegetable, stew vegetables, eggplant, green squash, local vegetables, pepper, mushroom, cucumber, garlic, kinds of cabbage, root vegetables, other vegetables
Legumes	Bean, chickpea, split pea, soybean, lentil, other cereals
Fried potato	Fried potato
Boiled potato	Boiled potato
Whole grains	Barbari bread, Sangak bread, Taftoon bread
Refined grains	Lavash bread, baguette, rice, macaroni
Snacks	Biscuits, puff, chips
Nuts	Peanut, almond, walnut, pistachio, hazelnut, roasted Seeds
Sweets and desert	Cakes, cookies, chocolate, pastry, dry sweet, honey, jam, halva
Sugar	Sugar, sugar cube, candy, sugar candy, tahini
Animal butter	Animal butter
Solid oil	Solid vegetable oil, animal fat, rump
Liquid oil	Liquid oil
Olive	Olive and olive oil
Mayonnaise	Mayonnaise
Soft drink	Carbonated drinks

**Assessment of other variables**

All participants were interviewed by trained interviewers to obtain necessary information including socio-demographic characteristics, family history of Cancer and CRC, physical activity, smoking habit, medical information (comorbidities, use of drugs and vitamin/mineral supplements) and cooking techniques. The weight of each individual with the least amount of clothing and a sensitivity of 100 grams by digital scale and height without shoes with a sensitivity of 0.1 cm were measured. Physical activity was assessed by a validated questionnaire (22). Participants were asked to rate their daily activities such as walking, exercise, sleep, hours devoted to watching television, housework, bathing, etc., along with the intensity of the activity reported. Total activity was reported for 24 h and the metabolic equivalent of tasks were calculated based on these self-reports.

**Statistical analyses**

Data analysis was performed by IBM Statistical Package Software for Social Science (SPSS), version 21 (SPSS Inc., Chicago, IL, USA). Normality of the data was checked using Kolmogorov-Smirnov’s test. Baseline characteristics of participants were expressed as mean (SD) for quantitative variables, and frequency and percentages for qualitative variables. Comparison of baseline characteristics and dietary intakes between cases and controls were done using independent sample t-test or mann-whitny U test for continuous variables and Chi Square for categorical variables, respectively. To extract Dietary Patterns, Principal Component Analysis (PCA) based on the 29 food groups was applied. Varimax rotation was used for improving interpretation and minimizing correlation between the factors. Statistical correlation between variables and adequacy of sample size was tested, using the Bartlett test of sphericity and the Kaiser-Mayer-Olkin test. The selection of dietary patterns was done using scree plot (eigenvalue >1). Post-rotated factor loadings showed three dietary patterns described the sample and these patterns were labelled based on each food group having the highest loading on each pattern. Food groups with positive loadings in each pattern indicate the direct relationship with that pattern and food groups with negative loadings shows the inverse relationship with that pattern. The factor score for each pattern was calculated by summing the consumption of each food group that were weighted by factor loading and each person received an individual factor score for each identified pattern (23). Dietary patterns were categorized according to median of factor scores. Logistic regression was used to determine the odds ratio (OR) with 95% confidence interval (CI) of CRC and CRA by higher scores on the dietary patterns. All models were adjusted for potential confounding variables. The OR and 95% CI for CRC adjusted for age, family history of cancer, family history of CRC, physical activity and calcium supplement. Also, the OR and 95% CI for CRA adjusted for age, comorbidity, CRC family history, common ways of cooking food, physical activity and calcium supplement use. OR and 95 % confidence interval (CI) were reported, and P-values <0.05 were considered as statistically significant.

**Ethical approval**

The present study was approved by the Ethics Committee of Shahid Beheshti University of Medical Sciences. 

## Results

The socio-demographic and life style characteristics of 240 controls, 129 colorectal cancers and 130 colorectal adenomas were shown in table 2. By frequency matched design, cases and controls had the same age and sex distribution. There was no statistically significant difference between groups by BMI, Educational level, Smoking, Colorectal cancer family history in first degree, Vitamin D supplement and Energy intake. However, cancer cases were more likely to have family history of cancer in first degree than controls. On the other hand, compared to controls, Adenoma cases were more likely to have at least one comorbidity and higher intake of calcium supplement. Also there is a significant difference for common ways of cooking food between adenomas and controls group, adenomas commonly consume grilled and combined foods, while controls commonly consume boiled foods. Also, compared to cancers cases, controls significantly have higher physical activity. The results of Bartlett test of sphericity (P <0001) and the Kaiser-Mayer-Olkin test (0.64) have shown the suitability of using factor analysis for this study. Using factor analysis, three major dietary patterns were identified. The factor loading matrix for the three retained factors are presented in table 3. These three dietary pattern explained 25.07% of total variance in food intake. The first pattern with higher consumption of vegetables, tomato, carrot, fruits, fish, legumes, egg and poultry was named “Healthy” dietary pattern. The second pattern characterized by high intake of Sweets and desert, Snacks, High fat dairies, Red meat, Artificial juice, Mayonnaise, Nuts, processed meat, Fried potato and Animal butter labeled “westernized” dietary pattern. The third pattern which greatest loading on solid oil, tea, sugar, boiled potato and refined grain called “Traditional” dietary pattern. Table 4 and 5 present the odds ratio (OR) and their 95% confidence interval (CI) for CRC and CRA according to median of factor scores for the retained dietary patterns. After adjusting for confounders, the Healthy dietary pattern was associated with a decreased risk of CRC (OR=0.22, 95% CI=0.14-0.37) and CRA (OR=0.43, 95% CI=0.27-0.69). Higher intake of the Westernized pattern was positively associated with risk of CRC (OR=3.5, 95% CI=2.13-5.19) and CRA (OR=2.47, 95% CI=1.49-4.08). There was no significant association between Traditional pattern and the CRC (OR=99, 95% CI=0.61-1.59) and CRA (OR=0.85, 95% CI=0.54-1.35) risk.

**Table 2:** The main characteristics of the participant's study

Variables	Controls(n=240)	Cancers(n=129)	Adenomas(n=130)	P-value*	P-value†
Age(Mean ±SD)	55.08±9.45	56.6±11.5	56.46±10.01		
Gender (male) n(%)	133(55.4)	66(51.2)	59(45.4)		
BMI(Mean ±SD)	26.93±3.99	26.68±5.49	26.72±3.81	0.62	0.63
Residence n (%) Urban Rural	216(90.4) 20(8.4)	110(87.3) 16(12.7)	123(96.1) 5(3.9)	0.35	0.22
Educational level n(%) Illiterate Low education High education	31)13) 172(72) 34(14.2)	18(14.1) 94(73.4) 16(12.5)	14(10.9) 90(70.3) 24(18.8)	0.71	0.48
Smoking (yes) n(%)	42(17.5)	26(20.2)	27(20.8)	0.53	0.11
Comorbidity (yes) n(%)	41(17.1)	21(16.3)	40(30.8)	0.84	0.002
Family history of cancer in first degree (yes) n(%)	89(32.9)	66(51.2)	48(36.9)	0.001	0.43
Colorectal cancer family history in first degree (yes) n(%)	18(7.5)	10(7.8)	17(13.1)	0.15	0.08
Common ways of cooking food n(%) Fried Boiled Grilled Steam cook Combined	55(22.9) 81(33.8) 5(2.1) 3(1.3) 96(40)	40(31) 41(31.8) 0(0) 2(1.6) 46(35.7)	18(13.8) 34(26.2) 4(3.1) 0(0) 74(56.9)	0.25	0.01
Physical activity(Mean ±SD)(met/h/day)	40.06±9.87	36.61±15.11	38.54±9.39	0.008	0.14
Vitamin D supplement (yes) n(%)	56(23.3)	28(21.7)	40(30.8)	0.72	0.11
Calcium supplement (yes) n(%)	35(14.6)	28(21.7)	32(24.6)	0.08	0.01
Energy intake(Mean ±SD)	2367.42±673.1	2272.14±574.02	2303.6±669.9	0.17	0.38

‡ Matched variables of the study,

* p-value between cancers and controls,

† p-value between adenomas and controls independent sample t-test was used for continuous variables and Chi-square was used for categorical variables. MET: Metabolic equivalent

**Table 3:** Factor loading matrix of food groups for dietary patterns

Food group	Healthy pattern	Westernized pattern	Traditional pattern
Vegetables	0.771	.	.
Tomato	0.602	.	.
Carrot	0.559	.	.
Fruits	0.501	.	.
Fish	0.487	.	.
Legumes	0.438	.	.
Egg	0.400	.	.
Poultry	0.259	.	.
Sweets and desert	.	0.606	.
Snacks	.	0.548	.
Soft drinks	.	0.542	.
High fat dairies	.	0.478	0.296
Red meat	.	0.444	.
Artificial juice	.	0.394	.
Mayonnaise	.	0.394	.
Nuts	0.290	0.350	-0.302
Processed meat	.	0.328	.
Fried potato	.	0.221	.
Animal butter	.	0.216	.
Solid oil	.	0.311	0.726
Liquid oil	.	.	-0.676
Olive	0.324	.	-0.510
Low fat dairies	0.254	-0.313	-0.415
Tea	.	.	0.386
Sugar	.	0.237	0.311
Boiled potato	.	.	0.299
Refined grain	.	0.248	0.250
Coffee	.	.	-0.234
Total variance	9.51%	8.77%	6.79%

Estimated by a Principle Component Analysis (PCA) performed on 29 food group. Absolute factor loading values < 0.20 for both patterns were excluded for simplicity

**Table 4:** Odds ratios and 95% confidence intervals for colorectal cancer by higher scores on the dietary patterns (above median *vs *below median)

Dietary pattern	Cancers/Controls	OR^a^	95% CI	OR^b^	95% CI
Healthy pattern					
Low	93/91	1.00(Ref)		1.00(Ref)	
High	36/149	0.23	0.14-0.38	0.22	0.14-0.37
P for trend		<0.0001		<0.0001	
Westernized pattern					
Low	42/142	1.00(Ref)		1.00(Ref)	
High	87/98	3.6	2.23-5.83	3.5	2.13-5.19
P for trend		<0.0001		<0.0001	
Traditional pattern					
Low	67/117	1.00(Ref)		1.00(Ref)	
High	62/123	0.91	0.58-1.39	0.99	0.61-1.59
P for trend		0.62		0.97	

Logistic regression was performed to obtain the odds ratio (95% CI) of colorectal cancer;

a Age adjusted model.

b Additionally adjusted for cancer family history, CRC family history, physical activity and calcium supplement use.

**Table 5:** Odds ratios and 95% confidence intervals for colorectal adenomas according to dietary pattern scores (above median *vs *below median)

Dietary pattern	adenoma/Controls	OR^a^	95% CI	OR^b^	95% CI
Healthy pattern					
Low	81/104	1.00(Ref)		1.00(Ref)	
High	49/136	0.46	0.31-0.72	0.43	0.27-0.69
P for trend		<0.0001		<0.0001	
Westernized pattern					
Low	50/135	1.00(Ref)		1.00(Ref)	
High	80/105	2.4	1.53-3.86	2.47	1.49-4.08
P for trend		<0.0001		<0.0001	
Traditional pattern					
Low	71/114	1.00(Ref)		1.00(Ref)	
High	59/126	0.76	0.49-1.17	0.85	0.54-1.35
P for trend		0.52		0.51	

Logistic regression was performed to obtain the odds ratio (95% CI) of colorectal cancer;

a Age adjusted model.

b Additionally adjusted for comorbidity, CRC family history, common ways of cooking food, physical activity and calcium supplement use.

## Discussion

The findings of this case-control study reveal significant reductions in CRC and CRA risk with the “Healthy” dietary pattern, indicating those having high healthy dietary pattern (high consumption of healthy food) had lower CRC and CRA risk. While the “Western” dietary pattern was related to a greater risk of CRC and CRA, those consuming more western foods had higher risk of CRC and CRA. There was no association between “Traditional” dietary pattern and risk of both CRC and CRA. 

Our results in regarding the association of dietary pattern and CRC and CRA risk are comparable with those identified in previous studies. A study in Korean population suggested that a diet characterized by high consumption of fruit and vegetable is associated with a decreased risk of CRC (24), another study in north Carolina showed an inverse association between a dietary pattern rich in fruits and whole grains and risk of rectal cancer (25). Results from a large prospective cohort from US indicated that a dietary pattern with greatest loading on fruits, vegetables, fish and poultry was associated with risk of CRA (26). Other studies reported that the adoption of dietary pattern characterized by fruits, vegetables, fish, whole grains and legumes intake may decrease the risk of CRC and CRA (27-29). In our study, vegetables, fruits, fish and legumes have high load in Healthy dietary pattern. The rationale for the potential benefits of such food groups relies on their richness in natural compounds, such as vitamins, polyphenols, PUFA and dietary fiber, which may have a role in action against colorectal carcinogenesis. Antioxidant, vitamins and polyphenols may prevent oxidative DNA damage, enhance DNA repair, and inhibit carcinogen bio-activation (30, 31). Dietary fiber may exert anti-carcinogenic effects through a direct action on the gastrointestinal tract, by reducing transit time and the contact of carcinogens with the colonic mucosa, increasing the binding of carcinogens, increasing the production of short-chain fatty acids, and decreasing concentrations of secondary bile acids (32). PUFAs, specially n-3 have demonstrated anti-inflammatory and anti-neoplastic effects on CRC that may rely on the interference with cytokines and prostaglandin metabolism (33). Considering the increased risk of CRC and CRA, the representative food components of Western dietary pattern were found to be disadvantageous in terms of CRC and have been investigated in numerous epidemiological studies (34, 35). In a large cohort study from US, the western dietary pattern that characterized by red and process meat, high fat dairies, refined grains and sweets and desert was positively associated with risk of colon cancer and distal adenoma (36). Similarly, makambi *et al.* demonstrated the higher scores on the western dietary pattern with high loading on refined grain, high fat dairies, red and process meat, egg and sweets associated with a higher risk of CRA (17). In this study, consumption of sweets and desert, soft drink, high fat dairies and red meat were related to Western dietary pattern. The most likely mechanism for certain food groups such as sweets, snacks and soft drink and their relationship with CRC could be defined by their effects on body weight and obesity, which are important risk factor for CRC (37, 38). Some studies showed an inverse association between milk and milk product and risk of CRC (39), but this relationship could be influenced by fat content. Some studies have shown that high fat dairies increased the risk of CRC (40). High fat consumption increased the concentration of bile acid which can promote CRC (41). In line with our results, other studies indicated that high intake of red or process meat increase the risk of CRC (42, 43). Previous studies suggested that carcinogenesis effect of red or process meat could be due to cooking and preservation method of meats and meats content such as saturated fatty acids, heme iron, heterocyclic amines (HCAs), polycyclic aromatic hydrocarbons (PAHs), malondialdehyde, nitrites and nitrates (44).

Also, we found that intake of Nuts is related to Western dietary pattern. Lee *et al.* reported that high intake of nut consumption associated with reduced risk of CRC(45), while Yang and colleagues failed to show any relationship between nut intake and risk of CRC (46). It may be due to the Iranian culture. In Iran, the consumption of nuts and sweets is common in feasts and usually consume in high salt content. It should be noted that in our study, sweets and desert had higher factor loading in Western dietary pattern. We found no significant association between traditional pattern and risk of both CRC and CRA. In contrast with our results, previous study conducted in Tabriz, a northwestern city of Iran, showed that the Iranian dietary pattern characterized by high consumption of tea, sugar, refined grain and solid oil was associated with increased risk of CRC (15), the difference between our results and this study could be due to study area. Tehran is the capital of Iran, and the hospitals we gathered our samples from, are referral hospitals that have patients from all over the country. Similar to this study (15), in our study tea and sugar were related to traditional dietary pattern. In Iran, tea especially black tea is the most popular drink that is commonly served with sugar or cube sugar. Findings on the relationship between tea consumption and risk of CRC are inconsistent. Some studies reported tea consumption is protective against CRC risk (47), while in other studies a positive association between tea and risk of CRC was observed (36, 48).

The strength of our study includes using validated instruments and ability to control for several potential confounding factors. This is the first study in the Middle-Eastern population that compared the CRC and CRA group with control group. Studies in developing countries can provide unique opportunities to test the association between diet and cancer. These countries are likely to have uniquely different (and more expansive) ranges of dietary factors, as well as possible genetic differences in susceptibility that allow for testing the association between diet and disease.

Despite its strengths, several limitations are also inherent in the present study. Measurement error is a limitation in any study of diet. Moreover, the possibility of selection and recall bias is difficult to avoid. However, use of new patient cases, using hospital controls and administering FFQs by trained interviewers in a hospital setting minimized these problems.

In conclusion, our study suggested that the “Healthy” dietary pattern seems reduce the risk of CRC and CRA, while the “Western” dietary pattern was associated with increased risk of both CRC and CRA.

## Conflict of interests

The authors declare that they have no conflict of interest.
